# Once daily versus three times daily mesalazine granules in active ulcerative colitis: a double-blind, double-dummy, randomised, non-inferiority trial

**DOI:** 10.1136/gut.2008.154302

**Published:** 2008-10-02

**Authors:** W Kruis, G Kiudelis, I Rácz, I A Gorelov, J Pokrotnieks, M Horynski, M Batovsky, J Kykal, S Boehm, R Greinwald, R Mueller

**Affiliations:** 1 Evangelisches Krankenhaus Kalk, University of Cologne, Cologne, Germany; 2 Kaunas University of Medicine Hospital, Kaunas, Lithuania; 3 Petz Aladár County and Teaching Hospital, Győr, Hungary; 4 Central Medical–Sanitary Hospital #122, Moscow, Russia; 5 Latvian Gastroenterology Center – Paula Stradina University Hospital, Riga, Latvia; 6 Centrum Medyczne “SOPMED” NZOZ, Sopot, Poland; 7 Derer’s University Hospital, Bratislava, Slovak Republic; 8 Hospital Ricany, Ricany, Czech Republic; 9 Dr Falk Pharma GmbH, Freiburg, Germany

## Abstract

**Objectives:**

To determine the therapeutic equivalence and safety of once daily (OD) versus three times daily (TID) dosing of a total daily dose of 3 g Salofalk (mesalazine) granules in patients with active ulcerative colitis.

**Design:**

A randomised, double-blind, double-dummy, parallel group, multicentre, international, phase III non-inferiority study.

**Setting:**

54 centres in 13 countries.

**Patients:**

380 patients with confirmed diagnosis of established or first attack of ulcerative colitis (clinical activity index (CAI)>4 and endoscopic index ⩾4 at baseline) were randomised and treated.

**Interventions:**

8-week treatment with either 3 g OD or 1 g TID mesalazine granules.

**Main outcome measures:**

Clinical remission (CAI⩽4) at study end.

**Results:**

380 patients were evaluable for efficacy and safety by intention-to-treat (ITT); 345 for per protocol (PP) analysis. In the ITT population, 79.1% in the OD group (n = 191) and 75.7% in the TID group (n = 189) achieved clinical remission (p<0.0001 for non-inferiority). Significantly more patients with proctosigmoiditis achieved clinical remission in the OD group (86%; n = 97) versus the TID group (73%; n = 100; p = 0.0298). About 70% of patients in both treatment groups achieved endoscopic remission, and 35% in the OD group and 41% in the TID group achieved histological remission. About 80% of all patients preferred OD dosing. Similar numbers of adverse events occurred in 55 patients (28.8%) in the OD group and in 61 patients (32.3%) in the TID group, indicating that the two dosing regimens were equally safe and well tolerated.

**Conclusions:**

OD 3 g mesalazine granules are as effective and safe as a TID 1 g schedule. With respect to the best possible adherence of patients to the treatment, OD dosing of mesalazine should be the preferred application mode in active ulcerative colitis.

**ClinicalTrials.gov Identifier:**

NCT00449722

Aminosalicylates (mesalazine, sulfasalazine, olsalazine, balsalazide) have been the mainstay of inflammatory bowel disease treatment for over 60 years.^1^ These drugs are prescribed for active disease as well as for relapse prevention in both ulcerative colitis and in Crohn’s disease. Aminosalicylates are the undisputed “gold standard” for maintaining remission in ulcerative colitis.^2^^–^ 4 Furthermore, they may have chemopreventive properties against colorectal cancer.^5^

Conventionally, a multiple daily dosing regimen of aminosalicylates is established. This is a demanding drug regimen, which can interfere with the everyday life of a patient, and can therefore reduce the quality of life. Moreover, the inconvenience of this dosing regimen can have a negative impact on adherence to the drugs, and thus, can lead to a poorer long-term prognosis. Adherence rates in prospective, community-based studies range from 40 to 60%,^6^ ^7^ and are particularly poor among patients in remission.^8^^–^ 11 Three times per day (TID) dosing of mesalazine was the most significant risk factor for partial non-compliance. A total of 57% of patients who were prescribed mesalazine TID admitted non-compliance.^10^ Moreover, it was shown that non-compliant patients had a 5-fold risk of experiencing a relapse as compared to patients taking more than 80% of their prescribed mesalazine medication.^12^ Thus, patients’ ideal therapy would be an effective, oral formulation with fewer tablets, less-frequent dosing, and minimal side effects.^13^

The dosing regimen of a therapeutic compound is usually based on its pharmacokinetic and pharmacodynamic properties such as systemic bioavailability as well as tissue peak and steady-state concentrations. However, aminosalicylates are thought to exert their therapeutic action topically from the luminal side of the intestines irrespective of their presence within the systemic circulation.^14^ To this end it may be possible to modify the dosing regimen to one more acceptable to the patient, particularly regarding compliance, and still maintain the therapeutic effect. In particular, this could involve decreasing the dosing frequency, and it has been observed that reducing dosing frequency improves compliance.^15^

Mesalazine, the therapeutically active moiety of sulfasalazine,^16^ ^17^ is routinely used in the treatment of ulcerative colitis. Salofalk granules are a multi-particulate formulation of mesalazine. Due to their enteric, acid-resistant film coating the dissolution starts approximately at pH⩾6.0, leading to a delayed and, due to the inner polymer matrix, prolonged release of the active ingredient throughout the entire colon.^18^ Salofalk granules are easy to swallow, are preferred by patients compared to enteric-coated tablets,^19^ ^20^ and 1 g TID has been proven to be the optimal dose for treatment of acute episodes of ulcerative colitis.^19^ The study aimed to prove that once daily (OD) dosing is at least as effective as a conventional TID dosing for the induction of remission in patients with active ulcerative colitis.

## MATERIAL AND METHODS

### Study population

Men and women aged 18–75 years with a histologically and endoscopically confirmed diagnosis of established or first attack of ulcerative colitis (clinical activity index (CAI)>4 and endoscopic index (EI)⩾4 at baseline, both according to Rachmilewitz^21^), and having an extent of disease >15 cm from the anus were entered in the study. Major exclusion criteria were Crohn’s disease, renal or liver insufficiency, baseline stool positive for bacteria causing bowel disease, immunosuppressants within 3 months and/or corticosteroids within 1 month prior to baseline, and current relapse that had occurred under maintenance treatment with >2 g/day mesalazine. All oral or rectal treatments for ulcerative colitis were to be stopped at baseline. All study participants gave their written informed consent prior to inclusion.

### Study design

This study was a randomised, double-blind, double-dummy, parallel group, multicentre clinical trial to evaluate the effect of dosing frequency of orally administered Salofalk granules (3 g OD vs 1 g TID) on the efficacy, safety and tolerability in patients with active ulcerative colitis. The study was performed according to a sequential adaptive design, with the first interim analysis planned to be performed after 200 intention-to-treat (ITT) evaluable patients had finished the trial. The planned total sample size was 320 patients. The study was conducted at 54 centres in 13 countries: Croatia, Czech Republic, Estonia, Germany, Hungary, Israel, Latvia, Lithuania, Poland, Russia, Slovak Republic, Slovenia and Ukraine. Patients were assigned to the treatment groups 1:1 based on a computer-generated randomisation scheme. The study treatment lasted 8 weeks, with control visits at 2, 4 and 6 weeks. Patients were enrolled from July 2005 to April 2006. A sponsor-independent data monitoring committee reviewed unblinded data of the interim analysis.

### Objectives

The primary aim of this study was to prove the clinical non-inferiority of an OD dosing as compared to a conventional TID dosing for the induction of remission in patients with active ulcerative colitis. The secondary objective was to compare the safety and tolerability between the different dosing schedules.

### Experimental procedures

Visits were scheduled for days 0 (baseline), 14, 28, 42 and 56 (week 8, final visit). If patients discontinued the study prematurely, a full final visit was performed if possible. At baseline, patients were examined physically and their demographics and medical history were recorded. Vital signs, laboratory tests, including haematology, biochemistry and urinalysis, and clinical signs of ulcerative colitis were assessed at each visit. Baseline and final visits comprised, in addition, endoscopy and histology. In patients with an established diagnosis of ulcerative colitis, endoscopy was required at least up to the proximal margin of inflammation. In newly diagnosed patients, total colonoscopy was mandatory.

### Study medication

Salofalk (mesalazine, 5-aminosalicylic acid (5-ASA)) and placebo granules were manufactured by Dr Falk Pharma, Freiburg, Germany. To keep the study double-blinded, a double-dummy design was used, ie, patients in the OD group had to administer 3 g mesalazine in the morning and 1 g placebo both at noon and in the evening; whereas patients in the TID group had to administer 1 g mesalazine in the morning, at noon and in the evening and, additionally, 2 g placebo in the morning.

Concomitant medications involved in the treatment of ulcerative colitis were not allowed during the study, including steroids, antibiotics, immunosuppressants, non-steroidal anti-inflammatory drugs, other forms of aminosalicylates, loperamide, psyllium-containing drugs, or new onset of probiotics.

## OUTCOME MEASURES

### Primary efficacy variable

The primary endpoint was the percentage of patients achieving clinical remission defined as a CAI⩽4 at the end of the study (with the “last observation carried forward” (LOCF) approach) in each of the two groups. The well-established CAI was calculated as the sum of the scores of seven variables (number of weekly stools, bloody stools, abdominal pain, general well-being, body temperature, extra-intestinal manifestations, erythrocyte sedimentation rate/haemoglobin).^21^ The scores for the first four variables were based on data collected in the patient’s diary during the 7 days preceding a study visit. Patients had to complete a daily diary throughout the study, which comprised seven items: number of stools, number of bloody stools, degree of rectal bleeding, general well-being, abdominal pain, regular intake of the study medication, and concomitant therapy administered. Disease was classified as mild if baseline CAI was ⩽8 and moderate if CAI>8.

### Secondary efficacy variables

Clinical improvement was defined as a decrease in CAI by at least 1 point from baseline to the individual study end. Additional endpoint was the disease activity index (DAI),^22^ with a modification of the mucosal subscore according to the US Food and Drug Administration (FDA) recommendation, ie, mucosal friability moved from a score of 1 to a score of 2,^23^ and active disease was defined as a bleeding score of >0 and mucosal appearance score of ⩾2 at baseline, and remission defined as a bleeding score of 0 and mucosal appearance score of ⩽1. The endoscopic index (EI) was evaluated according to Rachmilewitz,^21^ with endoscopic remission defined as EI<4. Furthermore, for patients with a baseline modified DAI_mucosal_⩾2, mucosal healing at the final endoscopy was defined as a modified DAI_mucosal_⩽1. Biopsies were taken from at least the sigmoid and the rectum and investigated by a central pathologist who was also blinded to the treatment given. The histological index (HI) was determined according to Riley *et al* 24; the total HI was based on the most severely inflamed segment. Time to first resolution of clinical symptoms was defined as the time from baseline to the day when the patient recorded for the first time in his or her diary to have no more than three bowel movements, all without blood. The physician’s global assessment (PGA) was evaluated on a 6-point scale according to Hanauer *et al*.^6^

### Safety variables

The frequency of adverse events (AEs) and clinically relevant changes in laboratory parameters (including a renal safety assessment) and vital signs were assessed. Compliance with study medication was checked by counting the medication returned at the follow-up visits.

### Sample size and statistical methods

For proving therapeutic equivalence (non-inferiority) of OD vs TID treatment, a one-sided test hypothesis was used. The non-inferiority margin was predefined as −15% for the difference of the remission rates between treatments. Assuming remission rates of 65% under both treatments, a sample size of 160 patients in each treatment arm was calculated to achieve an 80% power to yield a statistical significant result. As the study was conducted using a three-stage group sequential test design,^25^ ^26^ the boundary p value at the first interim analysis was given as p_1_ = 0.0048; thus the overall type I error rate of α = 0.025 (one-sided) was maintained.^27^ For confirmative proof of non-inferiority, the rate of clinical remission was tested using a χ^2^ test with maximum likelihood estimation according to Farrington and Manning,^28^ and differences between the remission rates and corresponding 97.5% one-sided repeated confidence intervals (CIs) were provided.^29^ The confirmatory test was based on the per-protocol (PP) analysis set. In addition, a sensitivity analysis was performed in the ITT population (ie, all randomised patients who had received the study medication). All other group comparisons were of an exploratory nature. For evaluation of secondary efficacy end points, 95% CIs were calculated for the differences between the two treatment groups OD vs TID. The median time to first symptomatic remission, in days, and the corresponding 95% CI was calculated for each treatment group using the Kaplan–Meier estimation. Treatment groups were compared by calculating the hazard ratio and the corresponding 95% CI assuming proportional hazards.

## RESULTS

### Patient characteristics

**Figure 1:**
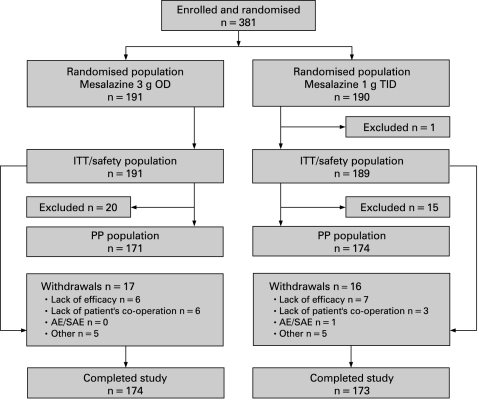
Patient disposition. One patient was excluded from the intention-to-treat population because he did not receive study medication. Patients were excluded from the per-protocol population because of protocol deviations. AEs, adverse events; ITT, intention-to-treat; OD, once daily; PP, per protocol; SAE, serious adverse event; TID, three times daily.

A total of 381 patients with ulcerative colitis (191 for the OD group, 190 for the TID group) were enrolled (fig 1). One patient, randomised to the TID group, did not receive study medication and, thus, was excluded from all analyses sets.

A total of 347 (91%) patients completed the study at the 8-week endpoint (fig 1). The main reasons for discontinuation of the treatment in these 33 patients were: lack of efficacy (6/17, 3% OD group; 7/16, 4% TID group); protocol violation (6/17, 3% OD group; 3/16, 2% TID group); adverse event (1/17, 0.5% TID group, baseline histology sample showed signs of amyloidosis); and other reasons (3% in each group).

There were no statistically significant differences between the treatment groups regarding the demographic and baseline disease characteristics, or pre-study maintenance medication (see table 1). Approximately three-quarters of the patients had established disease; half of the patients had distal ulcerative colitis, and two-thirds had mild disease. Only two-thirds of the patients with relapsing disease received maintenance medication for ulcerative colitis prior to enrolment, therefore oral mesalazine and sulfasalazine in mean dosages corresponding to less than or equal to 2 g mesalazine/day were the most common treatments. There were no differences between patients receiving maintenance treatment or not with regard to the distribution of their disease localisation.

**Table 1:** Patient baseline characteristics (intention-to-treat)

	Mesalazine, 3 g once daily (n = 191)	Mesalazine, 1 g three times daily (n = 189)	p Value
Male, n (%)	94 (49.2)	96 (50.8)	0.7582†
Mean (SD) age, years	41.8 (14.0)	43.3 (13.8)	0.2896‡
Mean body mass index (SD)	24.8 (4.6)	25.0 (4.3)	0.7599‡
Caucasian, n (%)	189 (99.0)	189 (100.0)	0.1584‡
Smoking status, n (%)			0.9781†
Current smoker	18 (9.4)	19 (10.1)	
Non-smoker	133 (69.6)	131 (69.3)	
Former smoker	40 (20.9)	39 (20.6)	
Diagnosis, n (%)			0.8618†
New diagnosis	50 (26.2)	48 (25.4)	
Established disease	141 (73.8)	141 (74.6)	
Median time since diagnosis, years (range)	2.8 (0.1–36.5)	3.1 (0.0–34.2)	0.3915§
Course of the established disease, n (%)			0.5552†
Chronically active disease	5 (3.5) (n = 141)	7 (5.0) (n = 141)	
Relapsing disease	136 (96.5) (n = 141)	134 (95.0) (n = 141)	
Mean number of previous episodes/relapses (relapsing disease) (SD)	4.3 (5.2) (n = 135)	5.1 (5.2) (n = 132)	0.2004‡
Median duration of present acute episode (relapsing disease), days (range)	27.0 (2–428) (n = 136)	29.5 (2–275) (n = 134)	0.5385§
Disease localisation, n (%)			0.1703†
Distal disease	97 (50.8%)	100 (52.9%)	
Proctosigmoiditis			
Proximal disease	94 (49.2%)	89 (47.1%)	
Left-sided colitis	55 (28.8%)	40 (21.2%)	
Subtotal-/pancolitis	39 (20.4%)	49 (25.9%)	
Mean length of inflammation, cm (SD)	49.1 (27.5) (n = 176)	49.5 (25.3) (n = 177)	0.8948‡
Mean disease activity (SD)			
Clinical activity index	8.1 (2.2)	7.9 (2.2)	0.5593‡
Modified disease activity index	7.4 (1.7)	7.0 (1.7)	0.0356‡
Endoscopic index	7.5 (1.9)	7.4 (1.9)	0.5149‡
Disease severity, n (%)			0.5697†
Mild (CAI⩽8)	121 (63.4)	125 (66.1)	
Moderate (CAI>8)	70 (36.6)	64 (33.9)	
Pre-study maintenance medication*, n (%)	94 (69.1) (n = 136)	89 (66.4) (n = 134)	0.6351†
Oral 5-aminosalicylate	59 (43.4)	53 (39.6)	
Oral sulfasalazine	26 (19.1)	26 (19.4)	
Rectal 5-aminosalicylate	10 (7.4)	9 (6.7)	
Immunosuppressants	3 (2.2)	1 (0.7)	
Oral corticosteroids	2 (1.5)	1 (0.7)	

Disease activity index with modified mucosal subscore (mucosal friability moved from a score of 1 to a score of 2).

*Doses of the pre-study medication did not violate the exclusion criterion. †χ^2^ test, two-sided. ‡t test, two-sided. §Wilcoxon rank-sum test, t approximation, two-sided.

CAI, clinical activity index; SD, standard deviation.

### Clinical effects

#### Primary efficacy endpoint

**Figure 2:**
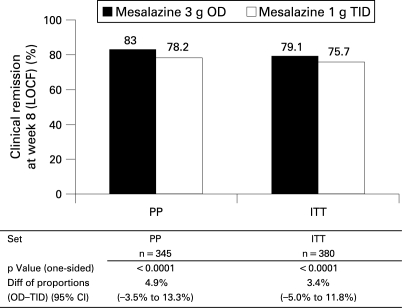
Clinical remission rates (CAI⩽4) at week 8 (LOCF). χ^2^ test (one-sided) for proving non-inferiority of OD vs TID treatment, with a pre-defined non-inferiority margin of −15% for the difference of the remission rates between treatments. CAI, clinical activity index; CI, confidence interval; ITT, intention-to-treat; LOCF, last observation carried forward; OD, once daily; PP, per protocol; TID, three times daily.

In the ITT population, 151/191 patients (79.1%) in the OD group and 143/189 patients (75.7%) in the TID group achieved clinical remission. Therefore, non-inferiority between the OD and TID group was concluded with a highly significant p value (p<0.0001) and a very tight 95% CI for the difference between both treatment groups (3.4% (−5.0% to 11.8%)). The results for the PP population were completely in line with those observed in the ITT population and confirmed the therapeutic equivalence between both treatment schedules (fig 2).

#### Influence of covariates on primary efficacy endpoint

The predefined exploratory subgroup analyses of the primary endpoint are presented for the ITT population in table 2. Evaluation clinical remission rates with regard to gender or disease duration (⩽5 years vs >5 years) did not show any statistically significant differences between and within each of the groups. With respect to baseline severity, there was no difference in the remission rates between the OD and TID group. However, within the OD group significantly more patients with mild as compared to moderate disease achieved clinical remission (85% vs 69%; p = 0.0067; χ^2^ test, two-sided). Disease localisation also had an impact on the remission rates achieved. Whereas no significant difference in proximal disease (ie, left-sided, subtotal, pancolitis) was observed between the OD and TID groups, there was a significant difference in distal disease between the groups (86% vs 73%; p = 0.0298; χ^2^ test, two-sided) as well as within the OD group itself between distal and proximal disease (86% vs 72%; p = 0.0247; χ^2^ test, two-sided). Previous maintenance treatment with up to 2 g mesalazine had no impact on the outcome compared to patients who did not receive maintenance therapy (OD 78% vs 81%, p = 0.6649; TID 72% vs 80%, p = 0.3094; χ^2^ test, two-sided).

**Table 2:** Clinical remission rates (CAI) by baseline covariates (intention-to-treat)

	Number (%) of patients in clinical remission (CAI⩽4) at final visit (LOCF)			
	Mesalazine 3 g OD (n = 191)	Mesalazine 1 g TID (n = 189)	Difference (%) OD − TID (95% CI)	p Value, χ^2^ (two-sided)
Gender				
Male	76/94 (81%)	76/96 (79%)	1.6 (−9.7 to 13.1)	0.7717
Female	75/97 (77%)	67/93 (72%)	5.3 (−7.1 to 17.6)	0.4027
Disease duration				
⩽5 years	95/121 (79%)	87/116 (75%)	3.5 (−7.2 to 14.3)	0.5220
>5 years	56/70 (80%)	56/73 (77%)	3.3 (−10.2 to 16.8)	0.6334
Severity (CAI at baseline)				
⩽8 points (mild disease)	103/121 (85%)*	99/125 (79%)	5.9 (−3.6 to 15.5)	0.2255
>8 points (moderate disease)	48/70 (69%)	44/64 (69%)	−0.2 (−15.9 to 15.5)	0.9822
Disease location				
Distal disease	83/97 (86%)†	73/100 (73%)	12.6 (1.4 to 23.7)	0.0298
Proximal disease	68/94 (72%)	70/89 (79%)	−6.3 (−18.7 to 6.1)	0.3217

Distal disease: proctosigmoiditis.

Proximal disease: left-sided ulcerative colitis/subtotal-/pancolitis.

*The difference (95%) in remission (mild vs moderate) within the OD group was 16.6% (4.0 to 29.1) (p = 0.0067).

†The difference (95%) in remission (proctosigmoiditis vs left-sided/pancolitis) within the OD group was 13.2% (1.8 to 24.7) (p = 0.0247).

CAI, clinical activity index; CI, confidence interval; LOCF, last observation carried forward; OD, once daily; TID, three times daily.

#### Secondary efficacy endpoints

All secondary efficacy endpoints were in line with the primary endpoint and supported the conclusion of therapeutic equivalence between the OD and TID groups. The results of the ITT population presented below were almost identical to those observed in the PP population.

#### Clinical activity index

**Figure 3:**
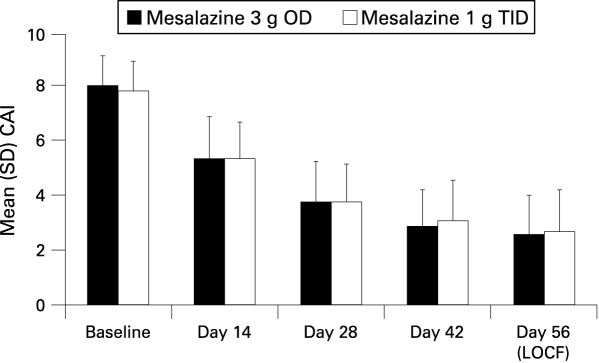
Course of the mean clinical activity index during the study (ITT). CAI, clinical activity index; ITT, intention-to-treat; LOCF, last observation carried forward; OD, once daily; SD, standard deviation; TID, three times daily.

In addition to clinical remission, approximately a further 13–15% of the patients experienced clinical improvement with no difference between groups (ITT). Clinically relevant sub-scores, ie, number of stools and number of bloody stools improved equally well in both groups; 107/191 (56%) and 109/189 (58%) of the patients in the OD and TID groups, respectively, had complete normal stool frequency, and 125/191 (65%) and 125/189 (66%) had no bloody stool at the end of the study. The time course of the CAI over the study period, as shown in fig 3, as well as the mean (SD) change from baseline in the CAI (OD, −5.5 (3.1); TID, −5.2 (3.4); p = 0.4360, t test) did not reveal any differences between the groups.

#### Time to first resolution of symptoms

The median time to first resolution of symptoms (ie, ⩽3 stools/day and free of blood) was 12 and 16 days in the OD and TID group, respectively (ITT). The hazard ratio of 0.961 together with a 95% CI of 0.771 to 1.197 indicated no clinically significant differences between the groups.

#### Modified disease activity index

As a secondary efficacy endpoint, the modified DAI score was used. Remission, defined according to the FDA recommendation as a bleeding score of 0 and mucosal appearance score of ⩽1, was obtained in 128/191 patients (67%) in the OD group and 122/187 patients (65.2%) in the TID group, respectively (ITT), with a difference in the proportion (95% CI) between the OD and TID groups of 1.8% (−7.8% to 11.3%).

#### Endoscopy and histology

Endoscopic remission using the EI was obtained in 135/191 patients (71%) in the OD group and 132/189 (70%) in the TID group at the end of the study (ITT). For patients with a baseline modified DAI_mucosal_⩾2, mucosal healing was obtained in 136/182 patients (75%) in the OD group and 129/177 (73%) in the TID group at the end of the study (ITT), with a difference in the proportion (95% CI) between the OD and TID groups of 1.8% (−7.3% to 10.9%). There was no significant difference between these values, and no significant difference in EI was observed between the two groups for the course of the study.

Histological remission was observed in 67/191 patients (35%) in the OD group and 77/189 patients (41%) in the TID group. Improvement was observed in another 46 patients (24%) in the OD group and 33 (17%) in the TID group. There was no statistically significant difference between the groups.

#### Physician’s global assessment

Therapeutic success, ie, at least a marked improvement in symptoms as rated by the PGA, was observed in 145/191 patients (76%) in the OD group and in 140/189 (74%) in the TID group. Therapeutic benefit of treatment, ie, at least slight improvement of symptoms, was observed in 176/191 patients (92%) in the OD group and 168/189 (89%) in the TID group. There were no differences between the groups.

#### Dosing preference of the study medication

Patients were asked which kind of dosing schedule they would prefer. The vast majority of all patients 313/380 (82%) preferred an OD dosing regimen; only 6/380 patients (2%) preferred the TID schedule, and 55/380 (14%) had no preference. For 6/380 patients (2%) no data were available.

### Safety and tolerability

A total of 78 treatment-emergent adverse events (TEAEs) occurred in 55/191 patients (29%) taking 3 g mesalazine OD and 83 TEAEs occurred in 61/189 patients (32%) taking 1 g mesalazine TID. The most common TEAEs were headache, worsening of ulcerative colitis, and nasopharyngitis (table 3). A causal relationship with study drug was considered as at least possible for eight TEAEs in 6/191 patients, (3%) in the OD group and for 10 TEAEs in 9/189 patients (5%) in the TID group. The vast majority of patients with adverse events experienced TEAEs of mild (OD, 43/55 patients (78%); TID, 47/61 patients (77%)), or moderate intensity (OD, 14/55 patients (25%); TID, 16/61 patients (26%)). Adverse events of severe intensity occurred only in seven patients of the OD group and in three patients of the TID group (most often “colitis aggravated”). In total, seven serious adverse events (SAEs) occurred in six patients (OD, four patients; TID, two patients). None of these SAEs was related to the study drug. No deaths occurred during this study. Nine AEs occurring in seven patients of the OD group, and seven AEs in seven patients of the TID group led to withdrawal of the study drug, with “deterioration of ulcerative colitis” as the most frequent reason for withdrawal (five patients in each group).

**Table 3:** Treatment-emergent adverse events

	3 g mesalazine OD (n = 191)	1 g mesalazine TID (n = 189)
Any TEAE	55 (28.8)	61 (32.3)
Any potential treatment-related AE	6 (3.1)	9 (4.8)
AE that led to withdrawal	7 (3.7)	7 (3.7)
Most frequent TEAEs occurring in more than 3% of patients		
Headache	9 (4.7)	15 (7.9)
Deterioration of ulcerative colitis	8 (4.2)	10 (5.3)
Nasopharyngitis	6 (3.1)	8 (4.2)
Any SAE	4 (2.1)	2 (1.1)
Individual SAEs		
Ulcerative colitis	4 (2.1)*	1 (0.5)
Viral upper respiratory tract infection	1 (0.5)*	0
Measles	0	1 (0.5)

Results are given as the number (%) of patients experiencing at least one treatment-emergent adverse event (TEAE).

*One patient in the OD group experienced both SAEs.

AE, adverse event; OD, once daily; SAE, serious adverse event; TID, three times daily.

Overall, no clinically relevant trends were observed in the course of the laboratory parameters. Urinary function tests using sensitive early markers of renal disease (α_1_-microglobulin, β-*N*-acetyl-d-glucosaminidase (β-NAG), cystatin C) showed no impairment of renal function, and indicated that an oral OD dose of 3 g mesalazine, which is associated with higher peak-plasma levels as compared to a 1 g TID regimen, is at least as safe as a 1 g TID dose with regard to potential tubulo-toxicity. On the contrary, decreases in these parameters even indicate an improvement of tubular function.

## DISCUSSION

The aim of the present study was to compare the efficacy and safety of two dosing regimens of the mesalazine preparation Salofalk granules in inducing remission in mild to moderate active ulcerative colitis. The total daily dose of 3 g was either given once a day (OD) or in divided portions three times per day (TID).

Both dosing regimens showed similar therapeutic effects, with 79% and 76% of the patients in the OD and TID groups, respectively, achieving clinical remission (ITT), which confirms the efficacy of mesalazine granules for the treatment of ulcerative colitis observed in previous randomised controlled trials.^19^ ^20^ The fact that very similar results were observed in the PP and ITT populations, and that the lower boundaries of the 95% CI for the difference between OD and TID were −3.5% (PP) and −5.0% (ITT), indicated that the conclusion of non-inferiority of 3 g OD vs 1 g TID is based on robust data. Moreover, it is of interest that nearly all values, although not statistically significantly different, favoured the OD dosing.

So far, only two other confirmative phase III studies have investigated the therapeutic efficacy of OD dosing of mesalazine in active ulcerative colitis,^30^ ^31^ and were recently summarised in a combined analysis.^23^ In order to compare the efficacy results between these studies and the current one, we used the same definition for remission in a post-hoc analysis as was used in these MMX mesalazine trials,^23^ ie, total modified DAI score ⩽1, with scores of zero for rectal bleeding and stool frequency, a combined PGA and sigmoidoscopy score of 1 or less, no friability, and at least a 1-point reduction from baseline in the sigmoidoscopy score. Using this very stringent definition, 70/191 patients (37%) in the OD group and 73/189 (39%) in the TID Salofalk granules group achieved remission (ITT population). These rates were nearly identical to those reported in the pooled analysis for 2.4 g/day (64/172 (37%)) and 4.8 g/day MMX mesalazine (61/174 (35%)), respectively.

There were no differences between the OD and TID groups with regard to influence of the remission rates by the duration of ulcerative colitis and baseline disease severity, although patients with mild ulcerative colitis showed better remission rates than those with moderate disease. Of interest is the significantly greater therapeutic effect of the OD dosing in proctosigmoiditis patients. Consequently, it may be hypothesised that OD dosing leads to higher luminal peak concentrations, particularly in the distal colon and that an OD dosing of mesalamine granules is well suited for oral treatment of distal disease, and thus might also enhance a patient’s compliance to treatment, as most of the patients prefer the oral over the rectal route of administration.

Endoscopy and histology revealed no statistically significant differences between the two dosing regimens. Mucosal healing rates, defined as modified DAI_mucosal_⩽1 at week 8 (LOCF), were in both mesalazine granules OD and TID groups (75% and 73%, respectively) comparable to those observed under treatment with 2.4 g/day (58/84 patients (69%)) and 4.8 g/day (66/85 patients (78%)) MMX mesalazine,^30^ and thus, again pointed towards similar therapeutic efficacy between both formulations.

Here, as in many other studies of mesalazine, treatment with the study medication was well tolerated and there was no difference in the occurrence of adverse events between the two dosing regimens. The majority of adverse events were mild or moderate in intensity and no unexpected side effects occurred, including no adverse effects on renal function, which had detailed observation throughout the study. The safety profile of the OD dosing is supported by the findings from a pharmacokinetic trial which showed that there is no accumulation of mesalazine during the steady-state dosing with 3 g OD mesalazine granules (arithmetic mean (SD) of accumulation ratios of the area under the curve was 1.17 (0.45), and at the maximal concentration time point it was 1.14 (0.39)).^32^

Intervention on dosing frequency affects both adherence and clinical outcome.^33^ Motivation to follow the treatment schedule can be increased by more suitable drug formulations and user-friendly intake frequencies. Indeed, in this study the vast majority of patients preferred a once-daily treatment schedule, and a content of 1.5 g mesalazine granules per sachet made it easy to take a total dose of 3 g per day. Non-adherence to the regimen of a prescribed medication is still a major and critical problem affecting the efficacy of a treatment, especially for long-term maintenance treatment. Kane showed that non-compliant patients had a 5-fold risk of relapses compared to patients taking more than 80% of their prescribed mesalazine medication.^12^ Moreover, Shale and Riley^10^ reported that approximately only half of patients adhere to the prescribed dosing schedule especially under a divided dosing regimen (three times daily), resulting in a median amount of medication administered of approximately 70% of the dose.

In conclusion, a 3 g once-daily dose of mesalazine granules (Salofalk granules) is at least as effective as a divided dose of 1 g given three times daily, and brings a substantial proportion of patients with mild-to-moderate active ulcerative colitis into clinical and endoscopic remission. Mesalazine granules demonstrated an excellent safety profile, independently of the mode of dosing. With respect to the best possible adherence of the patients to the treatment, once daily dosing of mesalazine should be the preferred mode of application in active ulcerative colitis.

## Appendix

The following were investigators in the International Salofalk OD Study Group.

**Croatia:** Mate Kozic, Ivo Klarin, Zvonimir Matas, Domagoj Kasun, Jadranko Turcinov, Roko Marcelic: Zadar.

**Czech Republic:** Bohumil Fixa, Tomas Vanasek: Hradec Kralove; Libor Gabalec, Vladimir Simon: Usti nad Orlici; Pavel Kohout: Prague; Jan Kykal: Ricany; Tomas Vich: Hranice.

**Estonia:** Karin Kull, Riina Salupere, Hele Remmel, Peeter Koiva, Katrin Labotkin: Tartu.

**Germany:** Wojtek Baniewicz, Susanne Bonnet, Bernd Vohmann: Mannheim; Wolfgang Kruis, Stephan Böhm: Cologne; Thomas Zeisler: Halle; Jürgen Zeus: Erlangen.

**Hungary:** János Banai, Tamás Szamosi, Zsófia Czeglédi: Budapest; Pál Demeter, Róbert Sike, József Penyige, Levente Bálint, Krisztina Sárdi, Ferenc Huoránszki: Budapest; Zoltan Döbrönte, Lilla Lakner, Zsolt Jakab: Szombathely; László Lakatos, Gábor Mester, Csolnoky Ferenc: Veszprém; István Rácz, Andrea Szabó, Gyula Pécsi, Mihály Csöndes, Hussam Saleh, Tibor Kárász: Györ; Zsolt Tulassay, László Herszényi, Anna Mária Németh, Pál Miheller: Budapest; László Újszászy, Andor Grenda: Miskolc; Borbala Velösy, Zsolt Virányi, Zsolt Dubravcsik: Kecskemét.

**Israel:** Simon Bar-Meir, Moshe Nadler: Tel Hashomer; Iris Dotan, Guy Rosner, Roman Grenshpon: Tel Aviv; Rami Eliakim, Irit Chermesh: Haifa; Mark Faszczyk, Arkady Berezovsky, Genadi Katz: Ashkelon; Dan Keret, Victoria Mindrul: Jerusalem; Alexandra Lavy, Dean Keren: Haifa; Ehud Melzer, Yuval Binder: Rechovot; Yaron Niv, Gerald Martin Fraser, Ebrahim Zebeede, Eyal Gal: Petach Tikva.

**Latvia:** Jelena Derova, Aleksejs Derovs: Riga; Juris Pokrotnieks, Aldis Pukitis, Mairita Ergle: Riga.

**Lithuania:** Gitana Acute, Violeta Vainoriute, Vytautas Metrikis, Algimantas Cepulis: Siauliai; Kestutis Adamonis, Arlandas Modzeliauskas: Kaunas; Audrone Buineviciute, Narimantas Samalavicius, Gintautas Radziunas: Vilnius; Gediminas Kiudelis, Limas Kupcinskas, Laimas Jonaitis: Kaunas; Giedrius Simulionis, Saulius Petrauskas, Valdas Kuolas: Klaipeda; Raimonda Vanagaitiene, Antanas Jablonskis, Ausra Navikiene: Vilnius.

**Poland:** Marek Horynski, Dariusz Kleczkowski: Sopot; Adam Kopon, Ewa Kopon: Torun; Krzysztof Marlicz, Halina Jaroszewicz-Heigelmann, Izabela Binkowska, Michal Wasilewicz: Szczecin; Aleksander Sieron, Wojciech Latos, Witold Zieleznik, Karolina Sieron-Stoltny: Bytom; Leszek Szczepanski, Robert Zwolak, Dariusz Chudzik, Mariusz Piotrowski, Marcin Mazurek: Lublin; Antoni Wysokinski, Romuald Grodzienski, Wojciech Kosikowski, Jaroslaw Drabko, Agnieszka Forys: Lublin.

**Russia:** Igor G. Bakulin, Vladislav G. Novozhenov, Marina A. Ivanova, Vladimir M. Ruseikin, Kerimulah D. Malabaev: Moscow; Elena A. Belousova, Tamara S. Mishurovskaya, Inna V. Nikulina: Moscow; Aleksander I. Gorelov, Elena A. Sishkova, Dmitry V. Raspereza, Tatiana V. Tinyakova: Saint-Petersburg; Vladimir B. Grinevich, Irina V. Gubonina, Anatoly M. Pershko: Saint-Petersburg; Tatyana L. Mikhailova, Oleg V. Golovenko, Ludmila A. Mayat, Pavel A. Makarchuk, Natalya S. Malakhova: Moscow; Emilia P. Yakovenko, Andrey V. Yakovenko, Natalya A. Agafonova, Antonina S. Pryanishnikova, Aleksandr N. Ivanov: Moscow; Konstantin P. Zhidkov, Oleg N. Bulavin, Fuza D. Albegova, Maria V. Vasilukova: Saint-Petersburg.

**Slovak Republic:** Boris Barický: Nitra; Marian Bátovský: Bratislava; Bozena Pekárková, Boris Pekarek: Trnava.

**Slovenia:** Milan Stefanovic, Zdravko Tosovic, Ljiljana Ljepovic: Bled.

**Ukraine:** Andrey Dorofeyev, Kira Lynevskaya, Olga Rassokhina: Donetsk; Yurii Siĺvestrovich Lozynsky, Maria Lozynska, Stanislav Golovchansky: Lvov; Mikhail Petrovich Zakharash, Yurii Zakharash, Tetyana Kravchenko: Kiev.
